# Supplementary material for: Multi-Year Retrospective Analysis of Mortality and Readmissions Correlated with STOPP/START and the American Geriatric Society Beers Criteria Applied to Calgary Hospital Admissions
Source: Geriatrics (Basel). 2023 Oct 9;8(5):100. doi: 10.3390/geriatrics8050100 (PMC10606166; doi:10.3390/geriatrics8050100)
Supplement: Supplementary file 1 [file geriatrics-08-00100-s001.zip › geriatrics-2582350-supplementary.pdf]

**Supplemental Table S1.** Numbers of medication by class prescribed to hospitalised Calgary seniors 2013-2021.

| Medication Class | Count |
|---|---|
| **Statin** | 2706992 |
| **PPI** | 2477437 |
| **Calciumblocker** | 2343022 |
| **Betablocker** | 2120849 |
| **Appropriatebetablocker*** | 1866600 |
| **Aceinhibitor** | 1786653 |
| **Antiplatelet** | 1724720 |
| **NSAID** | 1661178 |
| **Thyroxine** | 1537833 |
| **ARB** | 1363172 |
| **Aspirin** | 1317398 |
| **Opioid** | 1263059 |
| **Acetaminophen** | 1194105 |
| **SSRI** | 1076966 |
| **Loopdiuretic** | 996801 |
| **Metformin** | 962475 |
| **Antidepressantmore** | 915163 |
| **Hypnoticz** | 791259 |
| **A1blocker** | 727397 |
| **Anticholmusc** | 692128 |
| **Corticosystemic** | 678234 |
| **Neuroleptic** | 677408 |
| **Vitkantagonist** | 661224 |
| **Beta2agonist** | 582813 |
| **Laxative** | 574443 |
| **SNRI** | 563657 |
| **Hypocalcaemia** | 562393 |
| **Benzodiazepine** | 529618 |
| **Bisphosphonate** | 522139 |

**Supplemental Table S2.** First admission. Gender, age, comorbidities, numbers of medications, PIMs, PPOs, Charlson Index and correlations with readmission or mortality within 6 months of discharge (data for 129443 admissions).

| **Risk factor** | **Readmission within 6 months** | | | **Mortality within 6 months** | | |
|---|---|---|---|---|---|---|
| **Unadjusted ORs and 95%CIs** | | | | | | |
| | OR | 95%CI | p | OR | 95%CI | p |
| Gender | 1.17 | 1.14-1.20 | <.001 | 1.07 | 1.03-1.11 | <.001 |
| Age at admission | 1.01 | 1.01-1.02 | <.001 | 1.07 | 1.07-1.07 | <.001 |
| Number of comorbidities | 1.06 | 1.05-1.06 | <.001 | 1.21 | 1.20-1.21 | <.001 |
| Post admission Rx number | 1.13 | 1.12-1.13 | <.001 | 1.07 | 1.06-1.07 | <.001 |
| Total STOPP PIMs | 1.17 | 1.16-1.17 | <.001 | 1.04 | 1.03-1.05 | <.001 |
| START Omissions (PPOs) not corrected | 1.51 | 1.47-1.55 | <.001 | 2.47 | 2.57-2.56 | <.001 |
| START Omissions (PPOs) correctly prescribed | 1.43 | 1.39-1.47 | <.001 | 1.03 | 0.99-1.07 | .109 |
| AGS Beers PIMs | 1.12 | 1.11-1.12 | <.001 | 1.11 | 1.11-1.12 | <.001 |
| Charlson Index | 1.12 | 1.12-1.13 | <.001 | 1.46 | 1.45-1.47 | <.001 |
| **ORs and 95%CIs adjusted for age at admission, gender (male) and comorbidities** | | | | | | |
| | | | | | | |
| | OR | 95%CI | p | OR | 95%CI | p |
| Post admission Rx number | 1.12 | 1.12-1.12 | <.001 | 1.04 | 1.04-1.05 | <.001 |
| Total STOPP PIMs | 1.16 | 1.15-1.16 | <.001 | 0.99 | 0.96-1.00 | <.001 |

| | | | | | | |
|---|---|---|---|---|---|---|
| START PPOs not prescribed | 1.39 | 1.35-1.42 | <.001 | 1.56 | 1.50-1.63 | <.001 |
| START PPOS correctly prescribed | 1.26 | 1.23-1.30 | <.001 | 0.51 | 0.49-0.53 | <.001 |
| AGS Beers | 1.11 | 1.11-1.11 | <.001 | 1.08 | 1.07-1.08 | <.001 |

**Supplemental Table S3.** Second admission. Numbers of medications, PIMs, PPOs, and correlations with readmission or mortality within 6 months of discharge (data for 64441 admissions).

| Risk factor | Readmission within 6 months | | | Mortality within 6 months | | |
|---|---|---|---|---|---|---|
| **ORs and 95%CIs adjusted for age at admission, gender (male) and comorbidities** | | | | | | |
| | OR | 95%CI | p | OR | 95%CI | p |
| Post admission Rx number | 1.11 | 1.11-1.12 | <.001 | 1.01 | 1.1-1.02 | <.001 |
| Total STOPP PIMs | 1.15 | 1.14-1.15 | <.001 | 0.98 | 0.98-0.99 | <.001 |
| START PPOs not corrected | 1.41 | 1.36-1.46 | <.001 | 1.44 | 1.37-1.51 | <.001 |
| START PPOS correctly prescribed | 1.36 | 1.31-1.41 | <.001 | 0.52 | 0.50-0.55 | <.001 |
| AGS Beers | 1.10 | 1.10-1.11 | <.001 | 1.07 | 1.06-1.07 | <.001 |

**Supplemental Table S4.** Third admission. Numbers of medications, PIMs, PPOs, and correlations with readmission or mortality within 6 months of discharge (data for 35206 admissions).

| Risk factor | Readmission within 6 months | | | Mortality within 6 months | | |
|---|---|---|---|---|---|---|
| **ORs and 95%CIs adjusted for age at admission, gender (male) and comorbidities** | | | | | | |
| Post admission Rx number | 1.10 | 1.09-1.10 | <.001 | 1.00 | 0.99-1.01 | <.667 |
| Total STOPP PIMs | 1.14 | 1.13-1.15 | <.001 | 0.98 | 0.97-1.00 | <.004 |
| START PPOs not corrected | 1.41 | 1.35-1.48 | <.001 | 1.33 | 1.25-1.41 | <.001 |
| START PPOS correctly prescribed | 1.42 | 1.36-1.49 | <.001 | 1.04 | 1.04-1.04 | <.001 |
| AGS Beers | 1.09 | 1.08-1.10 | <.001 | 1.06 | 1.05-1.06 | <.001 |

**Supplemental Table S5.** Fourth admission. Numbers of medications, PIMs, PPOs, Charlson Index and correlations with readmission or mortality within 6 months of discharge (data for 20354 admissions).

| Risk factor | Readmission within 6 months | | | Mortality within 6 months | | |
|---|---|---|---|---|---|---|
| **ORs and 95%CIs adjusted for age at admission, gender (male) and comorbidities** | | | | | | |
| | OR | 95%CI | p | OR | 95%CI | p |
| Post admission Rx number | 1.10 | 1.09-1.10 | <.001 | 0.98 | 0.98-0.99 | <.001 |
| Total STOPP PIMs | 1.15 | 1.14-1.17 | <.001 | 0.96 | 0.95-0.98 | <.001 |
| START PPOs not corrected | 1.35 | 1.28-1.44 | <.001 | 1.30 | 1.21-1.40 | <.001 |
| START PPOS correctly prescribed | 1.50 | 1.41-1.59 | <.001 | 0.56 | 0.52-0.61 | <.001 |
| AGS Beers | 1.09 | 1.08-1.101 | <.001 | 1.04 | 1.03-1.05 | <.001 |

**Supplemental Table S6.** Fifth admission. Number of medications, PIMs, PPOs, Charlson Index and correlations with readmission or mortality within 6 months of discharge (data for 12271 admissions).

| Risk factor | Readmission within 6 months | | | Mortality within 6 months | | |
|---|---|---|---|---|---|---|
| **ORs and 95%CIs adjusted for age at admission, gender (male) and comorbidities** | | | | | | |
| | OR | 95%CI | p | OR | 95%CI | p |
| Post admission Rx number | 1.08 | 1.07-1.09 | <.001 | 0.97 | 0.96-0.98 | <.001 |
| Total STOPP PIMs | 1.13 | 1.12-1.15 | <.001 | 0.97 | 0.95-0.98 | <.001 |
| START PPOs not corrected | 1.38 | 1.28-1.49 | <.001 | 1.23 | 1.12-1.34 | <.001 |
| START PPOS correctly prescribed | 1.49 | 1.38-1.60 | <.001 | 0.63 | 0.57-0.68 | <.001 |
| AGS Beers | 1.07 | 1.06-1.09 | <.001 | 1.03 | 1.03-1.04 | <.001 |

**Supplemental Table S7.** Sixth admission. Numbers of medications, PIMs, PPOs, Charlson Index and correlations with readmission or mortality within 6 months of discharge (data for 7577 admissions).

| Risk factor | Readmission within 6 months | | | Mortality within 6 months | | |
|---|---|---|---|---|---|---|
| **ORs and 95%CIs adjusted for age at admission, gender (male) and comorbidities** | | | | | | |
| | OR | 95%CI | p | OR | 95%CI | p |
| Post admission Rx number | 1.08 | 1.07-1.09 | <.001 | 0.95 | 0.94-0.96 | <.001 |
| Total STOPP PIMs | 1.14 | 1.12-1.16 | <.001 | 0.97 | 0.95-0.99 | <.001 |
| START PPOs not corrected | 1.47 | 1.34-1.62 | <.001 | 1.24 | 1.11-1.40 | <.001 |
| START PPOS correctly prescribed | 1.64 | 1.49-1.81 | <.001 | 0.68 | 0.61-0.76 | <.001 |
| AGS Beers | 1.08 | 1.07-1.09 | 1<.001 | 1.02 | 1.01-1.04 | <.001 |

**Supplemental Table S8.** Seventh through 39th admissions. Numbers of medications, PIMs, PPOs, Charlson Index and correlations with readmission or mortality within 6 months of discharge (data for 10994 admissions).

| **Risk factor** | **Readmission within 6 months** | | | **Mortality within 6 months** | | |
|---|---|---|---|---|---|---|
| **ORs and 95%CIs adjusted for age at admission, gender (male) and comorbidities** | | | | | | |
| | OR | 95%CI | p | OR | 95%CI | p |
| Post admission Rx number | 1.06 | 1.06-1.07 | <.001 | 0.95 | 0.94-0.96 | <.001 |
| Total STOPP PIMs | 1.13 | 1.12-1.15 | <.001 | 0.94 | 0.93-0.96 | <.001 |
| START PPOs not corrected | 1.23 | 1.14-1.34 | <.001 | 1.14 | 1.04-1.26 | <.001 |
| START PPOS correctly prescribed | 1.53 | 1.41-1.65 | <.001 | 0.71 | 0.65-0.78 | <.001 |
| AGS Beers | 1.07 | 1.06-1.08 | <.001 | 1.00 | 0.99-1.01 | <.892 |

**Supplemental Table S9.** Population of Calgary ages 65 to 100+, Canadian Census 2022.

| **Population of Calgary 2022 Canadian Census** | | | | | | |
|---|---|---|---|---|---|---|
| | **Counts** | | | **Rates in total population** | | |
| Age | Total | Male | Female | Total | Male | Female |
| 65-69 | 62120 | 30410 | 31710 | 4.8 | 4.7 | 4.8 |
| 70-74 | 46495 | 22225 | 24270 | 3.6 | 3.4 | 3.7 |
| 75-79 | 28965 | 13485 | 15485 | 2.2 | 2.1 | 2.4 |
| 80-84 | 19405 | 8370 | 11035 | 1.5 | 1.3 | 1.7 |
| 85-89 | 12620 | 4995 | 7625 | 1.0 | 0.8 | 1.2 |
| 90-94 | 6225 | 2115 | 4110 | 0.5 | 0.3 | 0.6 |
| 96-99 | 1375 | 390 | 990 | 0.1 | 0.1 | 0.2 |
| ≥100 | 195 | 45 | 155 | 0.0 | 0.0 | 0.0 |
| Total | 177405 | 82035 | 95375 | 13.6 | 12.6 | 14.5 |

Source: https://www12.statcan.gc.ca/census-recensement/2021/dp-pd/prof/index.cfm?Lang=E.
